# Educational differences in healthcare use among survivors after breast, prostate, lung, and colon cancer – a SEQUEL cohort study

**DOI:** 10.1186/s12913-023-09683-2

**Published:** 2023-06-22

**Authors:** Anne Katrine Graudal Levinsen, Trille Kristina Kjaer, Thomas Maltesen, Erik Jakobsen, Ismail Gögenur, Michael Borre, Peer Christiansen, Robert Zachariae, Søren Laurberg, Peter Christensen, Niels Kroman, Signe Benzon Larsen, Thea Helene Degett, Lisbet Rosenkrantz Hölmich, Peter de Nully Brown, Christoffer Johansen, Susanne K. Kjær, Lau Caspar Thygesen, Susanne Oksbjerg Dalton

**Affiliations:** 1 Survivorship and Inequality in Cancer, Danish Cancer Institute, 49 Strandboulevarden, Copenhagen, 2100 Denmark; 2 Statistics and Data Analysis, Danish Cancer Institute, Copenhagen, Denmark; 3 Department of Thoracic surgery, Odense University hospital, Odense, Denmark; 4 Department of Surgery, Center for Surgical Science, Zealand University Hospital, Køge, Denmark; 5 Institute for Clinical Medicine, Copenhagen University, Copenhagen, Denmark; 6 Department of Urology, Aarhus University Hospital, Aarhus, Denmark; 7 Danish Breast Cancer Group Center and Clinic for Late Effects, Aarhus, Denmark; 8 Department of Plastic and Breast Surgery, Aarhus University Hospital, Aarhus, Denmark; 9 Department of Surgery, Danish Cancer Society Centre for Research on Survivorship and Late Adverse Effects After Cancer in the Pelvic Organs, Aarhus University Hospital, Aarhus, Denmark; 10 Department of Breast Surgery, Copenhagen University Hospital Herlev, Copenhagen, Denmark; 11 Danish Cancer Society, Copenhagen, Denmark; 12 Urological Research Unit, Department of Urology, Copenhagen University Hospital, Rigshospitalet, Copenhagen, Denmark; 13 Department of Plastic Surgery, Copenhagen University Hospital, Herlev and Gentofte, Denmark; 14 Department of Hematology, Rigshospitalet, Copenhagen University Hospital, Copenhagen, Denmark; 15 Cancer late effects, Rigshospitalet, University of Copenhagen, Copenhagen, Denmark; 16 Psychological Aspects of Cancer, Danish Cancer Institute, Copenhagen, Denmark; 17 Unit of Virus, Lifestyle and Genes, Danish Cancer Institute, Copenhagen, Denmark; 18 Department of Gynecology, Rigshospitalet, University of Copenhagen, Copenhagen, Denmark; 19 National Institute of Public Health, University of Southern Denmark, Copenhagen, Denmark; 20 Danish Research Center for Equality in Cancer, Department of Clinical Oncology & Palliative Care, Zealand University Hospital, Næstved, Denmark

**Keywords:** Healthcare use, Social inequality, Survivorship, Breast cancer, Prostate cancer, Lung cancer, Colon cancer

## Abstract

**Background:**

Many cancer survivors experience late effects after cancer. Comorbidity, health literacy, late effects, and help-seeking behavior may affect healthcare use and may differ among socioeconomic groups. We examined healthcare use among cancer survivors, compared with cancer-free individuals, and investigated educational differences in healthcare use among cancer survivors.

**Methods:**

A Danish cohort of 127,472 breast, prostate, lung, and colon cancer survivors from the national cancer databases, and 637,258 age- and sex-matched cancer-free individuals was established. Date of entry was 12 months after diagnosis/index date (for cancer-free individuals). Follow-up ended at death, emigration, new primary cancer, December 31st, 2018, or up to 10 years. Information about education and healthcare use, defined as the number of consultations with general practitioner (GP), private practicing specialists (PPS), hospital, and acute healthcare contacts 1–9 years after diagnosis/index date, was extracted from national registers. We used Poisson regression models to compare healthcare use between cancer survivors and cancer-free individuals, and to investigate the association between education and healthcare use among cancer survivors.

**Results:**

Cancer survivors had more GP, hospital, and acute healthcare contacts than cancer-free individuals, while the use of PPS were alike. One-to-four-year survivors with short compared to long education had more GP consultations (breast, rate ratios (RR) = 1.28, 95% CI = 1.25–1.30; prostate, RR = 1.14, 95% CI = 1.10–1.18; lung, RR = 1.18, 95% CI = 1.13–1.23; and colon cancer, RR = 1.17, 95% CI = 1.13–1.22) and acute contacts (breast, RR = 1.35, 95% CI = 1.26–1.45; prostate, RR = 1.26, 95% CI = 1.15–1.38; lung, RR = 1.24, 95% CI = 1.16–1.33; and colon cancer, RR = 1.35, 95% CI = 1.14–1.60), even after adjusting for comorbidity. One-to-four-year survivors with short compared to long education had less consultations with PPS, while no association was observed for hospital contacts.

**Conclusion:**

Cancer survivors used more healthcare than cancer-free individuals. Cancer survivors with short education had more GP and acute healthcare contacts than survivors with long education. To optimize healthcare use after cancer, we need to better understand survivors’ healthcare-seeking behaviors and their specific needs, especially among survivors with short education.

**Supplementary Information:**

The online version contains supplementary material available at 10.1186/s12913-023-09683-2.

## Introduction

The number of cancer survivors are growing worldwide, mainly because of ageing populations, earlier detection, and improved cancer treatments [1, 2]. With advances in treatment, a wide range of late effects is becoming apparent [2–5]. Physical and psychological late effects of cancer can be disabling, and some acute late effects, such as pain, may become chronic, while other late effects may occur years after end of treatment [2, 3, 6]. Late effects may contribute to a greater use of healthcare services among cancer survivors and the rising cancer survivor population is likely to increase the future workload for the healthcare sectors.

Previous studies have shown increased consultation rates in the primary healthcare sector among cancer survivors compared to cancer-free individuals for up to 6 years after diagnosis [7–13]. One study reported an increased number of contacts with medical specialists, outpatient clinics, and other doctors within the past 3 months among 5-15-year breast cancer survivors compared to a general population [4]. In European population-based studies, it has been shown that lower socioeconomic position (SEP) is associated with increased healthcare use [14, 15], and that long education is associated with fewer out-of-hour consultations with general practitioners (GPs) among chronically ill patients [16]. Only two studies have examined the association between SEP and healthcare use among breast or colorectal cancer survivors [4, 17], and to our knowledge, no other studies have explored the impact of SEP on healthcare use among cancer survivors. To better anticipate future developments in cancer survivors’ use of healthcare and socioeconomic differences in healthcare use among survivors from different cancers and with different clinical characteristics, population-based data is of increasing importance.

In the present prospective population-based cohort study, we investigated SEP-related differences in the use of elective and acute healthcare services in 1-9-year survivors of the most prevalent cancer types in Denmark (breast, prostate, lung, and colon cancer) [18]. We identified cancer survivors using the Danish SEQUEL cohort (‘inequality in SEQUELae after cancer’), which consists of comprehensive information from Danish national registers and clinical cancer databases. To quantify the overall healthcare use, we compared the use of GP consultations, consultations with private practicing medical specialists, hospital, and acute healthcare contacts between cancer survivors and cancer-free individuals. Within the cancer survivor population, we investigated whether education, a SEP proxy, is associated with healthcare use among cancer survivors, including whether systematic differences in healthcare use can be detected across socioeconomic groups of cancer survivors with different clinical characteristics such as stage at diagnosis, treatment, comorbidity, and time since diagnosis.

## Methods

### Study population

**Fig. 1:**
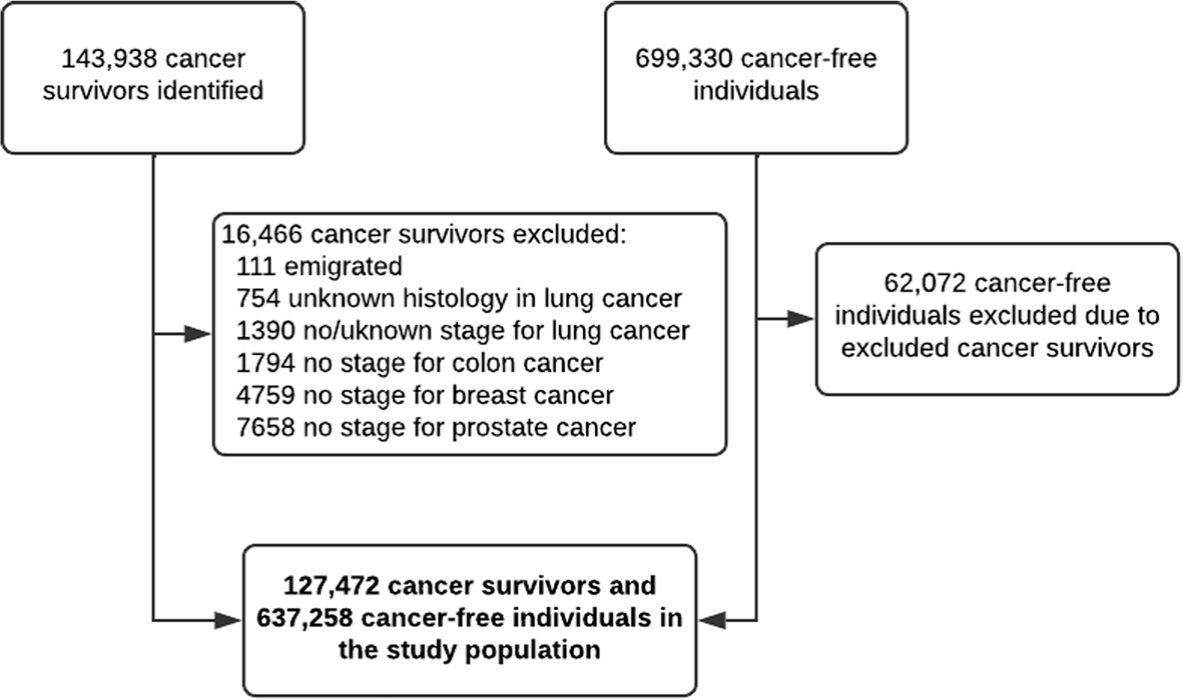
Population flowchart including 1-9-year Danish survivors of breast, prostate, lung, colon cancer, and cancer-free individuals

We identified 143,938 adult survivors after cancer of the breast (diagnosed from January 1997), prostate (diagnosed from January 2010), lung (diagnosed from January 2003), and colon (diagnosed from January 2005), as reported in their respective national clinical cancer databases: Danish Breast Cancer Cooperative Group Database [19], Danish Prostate Cancer Database [20], Danish Lung Cancer Registry [21], and Danish Colorectal Cancer Group Database [22]. To be eligible, survivors had to be 40 years or older at the time of diagnosis, residents in Denmark, have no previous cancer diagnosis (except non-melanoma skin cancer), and be alive at least 12 months after diagnosis (time of entry). We chose to define survivorship after cancer as starting from 12 months after diagnosis as most cancer patients will have finalized primary treatment by then. Individuals were excluded if they emigrated (*n* = 111), had unknown histology (only lung cancer) (*n* = 754), or had missing information on stage at diagnosis (*n* = 15,601) (Fig. 1). For each cancer survivor, we identified four to five individuals matched on age and sex at index date (date of diagnosis for the corresponding cancer survivor), who had no cancer diagnosis prior to the date of entry (12 months after index date). This resulted in the inclusion of 127,472 cancer survivors and 637,258 matched cancer-free individuals (Fig. 1).

Since 1968, all residents in Denmark have been provided with a unique identification number [23]. This number was used to obtain individual-level information on vital status and migrations during follow-up for both the cancer survivors and cancer-free individuals through linkage to the Central Population Registry (CPR) [24]. The cohort were followed from date of entry until the time of death, emigration, new primary cancer (for cancer survivors), a cancer diagnosis (for cancer-free individuals), December 31st, 2018, or up to 10 years, whichever came first. Non-melanoma skin cancer was not counted as a censoring event due to low morbidity and mortality.

### Socioeconomic factors

We used educational level as an indicator of SEP, as education reflects attained knowledge, skills, and may reflect an individuals’ understanding of health information [25]. Individual-level information about education was obtained from the Danish social registers administered by Statistics Denmark [23]. Educational level was measured as the highest attained education before cancer diagnosis/index date and categorized as short (mandatory school; ≤ 9 years), medium (upper secondary/high school or vocational education; 10–12 years), and long education (higher education; > 12 years). As an indicator of social support, we obtained information from Statistics Denmark on cohabitation status at time of diagnosis/index date, categorized as living with a partner (married, cohabiting with or without children) or living alone (single, divorced, widower/widow).

### Healthcare use

The Danish healthcare system is publicly funded, which ensures free and equal access to healthcare largely free of co-payments for all citizens [26]. The provision of health care is divided between GPs, municipalities (primary sector), and hospitals (secondary sector). GPs holds a gatekeeping function for accessing all elective consultations with medical private practicing specialists (PPS) (except ear-nose-throat and eye specialists) and elective hospital visits [27]. Healthcare use was measured from date of entry. Information on GP consultations and consultations with PPS were obtained from the Danish National Health Service Register [28], and included the number of daytime face-to-face, e-mail, and telephone consultations. Hospital contacts included information on the number of outpatient visits and hospital admissions obtained from the Danish National Patient Registry [29]. We excluded all hospital contacts registered as cancer-specific follow-up visits. Acute healthcare contacts included the number of out-of-hour GP consultations, acute hospital admissions, and emergency department visits from both registers [28, 29].

### Clinical factors

Information on cancer diagnosis, stage, and treatment received during the 12 months after diagnosis was obtained from the national clinical cancer databases [19–22]. Stage was categorized into local/regional or advanced stage and received treatment was categorized as curatively intended or palliative treatment (Supplementary Table 1). Information on comorbidity was obtained from the Danish National Patient Registry [29] and assessed as comorbid disorders present in the 3 years to 90 days prior to the cancer diagnosis/index date, to adjust for any pre-existing morbidities. The Charlson Comorbidity Index Score was calculated and categorized into a score of 0, 1, or ≥ 2 [30], modified however, by excluding the cancer in question.

### Statistical analysis

We plotted the mean cumulative number of GP consultations and hospital contacts during follow-up for survivors after each investigated cancer type and corresponding cancer-free individuals, stratified by education, to visually examine the differences in use of GP and hospital services. We used the Nelson-Aalen estimator [31] to allow the same individual to have several GP consultations and hospital contacts during follow-up. Poisson regression models with a sandwich variance estimator were used to investigate healthcare use among cancer survivors compared to the matched cancer-free individuals and to compute the associations between education and healthcare use among cancer survivors only. All analyses were split among short- and long-term survivors, e.g., 1–4 and 5–9 years after diagnosis/index date, respectively. Time since diagnosis/index date was the underlying time scale included by an offset and we adjusted for age (continuous), sex, year of diagnosis/index date, education, cohabitation status, comorbidity, with additional adjustment for stage at diagnosis for analyses of educational differences in healthcare use among cancer survivors. To investigate whether clinical factors modified the association between educational level and healthcare use, we further stratified analyses by disease stage, comorbidity, and treatment. All analyses were conducted in R (version 4.1.2) [32].

## Results

### Study population

For the 127,472 survivors after breast, prostate, lung, and colon cancer and 637,258 matched cancer-free individuals, most had medium education, were living with a partner, and had no comorbidity. Most cancer survivors were diagnosed with local/regional stage disease, except for lung cancer survivors, and most had received curatively intended treatment in the first 12 months after diagnosis, although many prostate and lung cancer survivors had received palliative treatment (Table 1.).

**Table 1:** Characteristic of 127,472 breast, prostate, lung, colon cancer survivors, and matched cancer-free individuals

**Characteristics**	**Cancer survivors**				**Cancer-free individuals (** ***n*** **=637,258)** n (%)
	**Breast (** ***n*** **=66,536)** n (%)	**Prostate** (*n*=19,963) n (%)	**Lung (** ***n*** **=18,625)** n (%)	**Colon (** ***n*** **=22,348)** n (%)	
ICD-code	C50	C61	C34	C18-19	-
Sex					
Men	-	19,963 (100)	9010 (48)	11,395 (51)	201,797 (32)
Women	66,536 (100)	-	9615 (52)	10,953 (49)	435,461 (68)
Age (years): mean (range)	62 (40-103)	69 (40-96)	67 (40-96)	70 (40-100)	66 (40-103)
Follow-up time (years): mean (range)	6.1 (0-10)	3.3 (0-8.0)	2.2 (0-10)	3.9 (0-10)	5.5 (0-10)
Education^a^					
Short	15,744 (24)	4370 (22)	6336 (34)	6400 (29)	173,351 (27)
Medium	30,894 (46)	10,269 (51)	9243 (50)	10,557 (47)	296,590 (47)
Long	16,810 (25)	4963 (25)	2572 (14)	4532 (20)	142,104 (22)
Missing	3088 (5)	361 (2)	474 (2)	859 (4)	25,213 (4)
Cohabitation^b^					
Living with a partner	42,759 (64)	15,541 (78)	11,630 (62)	14,342 (64)	416,322 (65)
Living alone	23,716 (36)	4396 (22)	6981 (38)	7986 (36)	220,466 (35)
Unknown	61 (0)	26 (0)	14 (0)	20 (0)	470 (0)
Comorbidity^c^					
0	60,512 (91)	17,324 (87)	14,709 (79)	18,766 (84)	558,376 (87)
1	4561 (7)	1881 (9)	2760 (15)	2415 (11)	55,097 (9)
≥2	1463 (2)	758 (4)	1156 (6)	1167 (5)	23,785 (4)
Stage at diagnosis^d^					-
Local/regional	65,282 (98)	17,680 (89)	8237 (44)	18,416 (82)	
Advanced	1254 (2)	2283 (11)	10,388 (56)	3932 (18)	
Treatment during 12 mo. after diagnosis^e^					-
Curative	65,282 (98)	10,021 (50)	11,231 (60)	20,113 (90)	
Palliative	1254 (2)	6358 (32)	6501 (35)	1893 (9)	
None	-	3577 (18)	893 (5)	323 (1)	
Missing	-	7 (0)	-	19 (0)	

^a^Education: categorized as short (mandatory school; ≤ 9 years), medium (secondary education or vocational education; 10-12 years), and long education (higher education; > 12 years)

^b^Cohabitation: living with others included all who were married, in a registered partnership or co-living with a partner. Living alone included all who were single, divorced, or widower/widow

^c^Comorbidity was calculated based on the Charlson Comorbidity Index

^d^Stage at diagnosis: local/regional stage defined as any tumor size, any number of positive lymph nodes and no metastasis (breast cancer); TNM stage with any T, any N, and no M (prostate cancer); TNM stage at IA, IB, IIA, IIB, or IIIA (lung cancer), UICC (8^th^ edition) stage I, II, or III (colon cancer). Advanced stage defined as distant metastasis (breast cancer); TNM stage with metastasis (prostate cancer); TNM stage IIIB, IIIC, IVA, or IVB (lung cancer); UICC (8^th^ edition) stage IV (colon cancer)

^e^Treatment: patients with local/regional stage disease were categorized as having had curative treatment (breast cancer); prostatectomy, active surveillance, or curative radiotherapy (prostate cancer); curative chemo- and/or radiotherapy, surgery, neo- and/or adjuvant therapy (lung cancer); surgery with curative intent (colon cancer). Palliative treatment defined as advanced stage disease (breast cancer); palliative radiotherapy, endocrine therapy, or watchful waiting (prostate cancer); palliative chemo- and/or radiotherapy, or other treatment with palliative intent (lung cancer); surgery with palliative intent, chemo- and/or radiotherapy (colon cancer)

### Healthcare use

Survivors after breast, prostate, lung, and colon cancer had more GP consultations and hospital contacts within 1–4 and 5–9 years after diagnosis than cancer-free individuals, with incidence rates per 100 person-years ranging from 1218 to 1961 for GP consultations and 542 to 1291 for hospital contacts among 1-4-year cancer survivors versus 1042 to 1175 and 209 to 276 among cancer-free individuals, respectively (Table 2). This corresponds to, for instance, 12 to 19 GP consultations per year for cancer survivors, and 10 to 12 GP consultations per year for cancer-free individuals. Correspondingly, in adjusted analyses, 1-4- and 5-9-year survivors had statistically significantly higher rate ratios (RRs) for GP consultations (RRs range: 1.18–1.74 and 1.09–1.44, respectively) and hospital contacts (RRs range: 2.15–4.86 and 1.51–2.29, respectively) (Table 2). Cancer survivors also had more acute healthcare contacts than cancer-free individuals up to 10 years after diagnosis, while the use of PPS were similar for cancer survivors and cancer-free individuals (Supplementary Table 2). Adjusted analyses showed statistically significantly higher RRs of acute healthcare contacts for 1-4- and 5-9-year survivors compared to cancer-free individuals (RRs range: 1.36–3.57 and 1.27–2.18, respectively), and statistically significantly higher RRs of consultations with PPS for 1-4-year breast cancer survivors (RR = 1.13) and 5-9-year breast, prostate, and colon cancer survivors compared to cancer-free individuals (RRs range: 1.05–1.11) (Supplementary Table 2).

**Table 2:** Results from Poisson regression analyses examining healthcare use between cancer survivors and cancer-free individuals

	**GP consultations**					
	**1–4 years**			**5–9 years** ^a^		
	Crude incidence per 100 P-Y	Model 1 RR (95% CI)	Model 2 RR (95% CI)	Crude incidence per 100 P-Y	Model 1 RR (95% CI)	Model 2 RR (95% CI)
Breast cancer^b^	1218	1.17 (1.16; 1.18)	1.18 (1.18; 1.19)	1223	1.11 (1.10; 1.12)	1.12 (1.11; 1.13)
Cancer-free individuals	1042	Ref.	Ref.	1110	Ref.	Ref.
Prostate cancer^b^	1346	1.29 (1.27; 1.30)	1.32 (1.30; 1.33)	1353	1.21 (1.19; 1.23)	1.23 (1.20; 1.25)
Cancer-free individuals	1050	Ref.	Ref.	1128	Ref.	Ref.
Lung cancer	1961	1.84 (1.81; 1.87)	1.74 (1.72; 1.77)	1670	1.50 (1.46; 1.54)	1.44 (1.40; 1.48)
Cancer-free individuals	1071	Ref.	Ref.	1143	Ref.	Ref.
Colon cancer	1415	1.20 (1.18; 1.21)	1.19 (1.18; 1.21)	1377	1.09 (1.07; 1.12)	1.09 (1.07; 1.11)
Cancer-free individuals	1175	Ref.	Ref.	1236	Ref.	Ref.
	**Hospital services**					
	**1–4 years**			**5–9 years** ^a^		
	Crude incidence per 100 P-Y	Model 1 RR (95% CI)	Model 2 RR (95% CI)	Crude incidence per 100 P-Y	Model 1 RR (95% CI)	Model 2 RR (95% CI)
Breast cancer^b^	542	2.60 (2.57; 2.63)	2.62 (2.59; 2.65)	427	1.86 (1.83; 1.90)	1.88 (1.85; 1.91)
Cancer-free individuals	209	Ref.	Ref.	229	Ref.	Ref.
Prostate cancer^b^	569	2.07 (2.03; 2.12)	2.15 (2.11; 2.20)	402	1.56 (1.50; 1.62)	1.59 (1.53; 1.65)
Cancer-free individuals	276	Ref.	Ref.	259	Ref.	Ref.
Lung cancer	1291	5.14 (5.03; 5.26)	4.86 (4.75; 4.98)	636	2.41 (2.30; 2.53)	2.29 (2.18; 2.41)
Cancer-free individuals	251	Ref.	Ref.	271	Ref.	Ref.
Colon cancer	666	2.46 (2.40; 2.52)	2.44 (2.38; 2.50)	427	1.52 (1.45; 1.59)	1.51 (1.44; 1.57)
Cancer-free individuals	270	Ref.	Ref.	280	Ref.	Ref.

Cancer-free matched comparison people are the reference for all analyses. *GP* general practitioner, *RR* rate ratios, *CI* confidence interval, *P-Y* person-years

Model 1: adjusted for age, sex, time since diagnosis/index date, year of diagnosis

Model 2: adjusted for age, sex, time since diagnosis/index date, year of diagnosis, cohabitation status, comorbidity, education

^a^5–8 year for survivors after prostate cancer

^b^Analyses are not adjusted for sex

### Educational differences in healthcare use

**Fig. 2:**
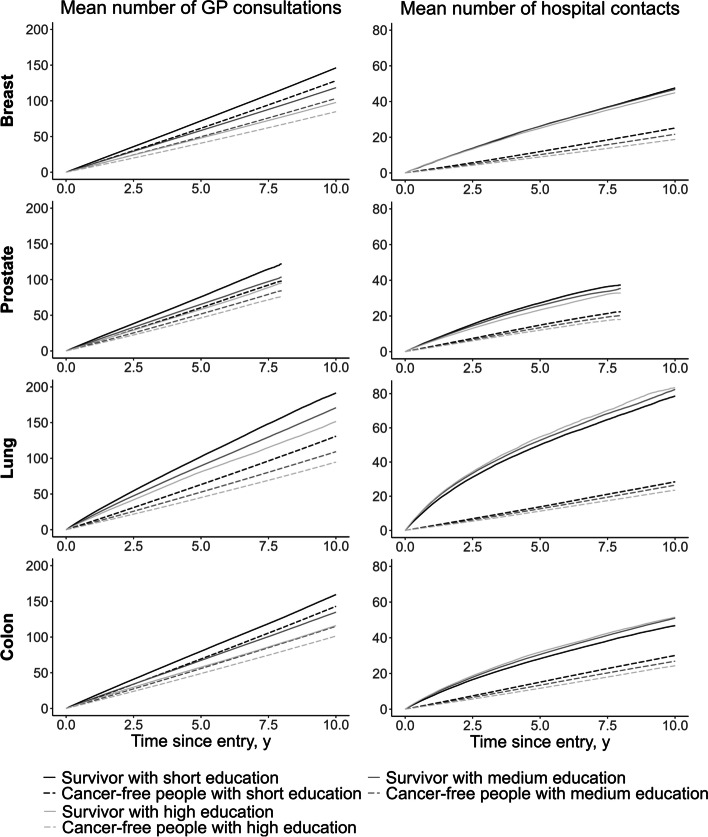
Mean cumulative number of GP consultations and hospital contacts for cancer survivors and cancer-free individuals. Note that x-axis is not the same

Regardless of cancer type, survivors with short education had more GP consultations than survivors with medium or long education. Similar educational differences in the mean cumulative number of GP consultations were observed among cancer-free individuals, while there were no statistically significant educational differences in the mean cumulative number of used hospital contacts among cancer survivors or cancer-free individuals (Fig. 2, confidence intervals are not shown).

**Fig. 3:**
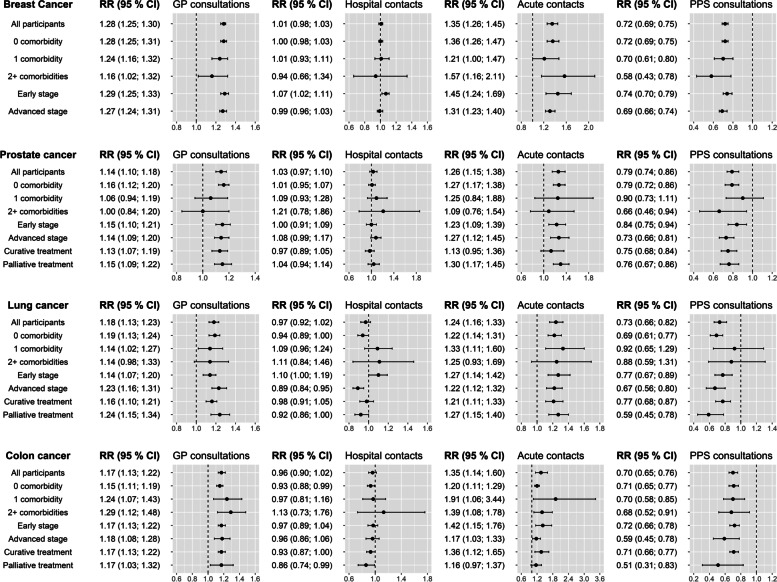
RRs of GP, hospital, acute, and PPS contacts among 1-4-year-CS with short versus long education. Long education is the reference for all analyses. All analyses are adjusted for age, sex (except prostate and breast cancer survivor populations), time since diagnosis, year of diagnosis, cohabitations status, comorbidity, and stage. RR, rate ratio; GP, general practitioner; PPS, private practicing specialist; CS, cancer survivors; CI, confidence intervals. Note that x-axis is not the same

In adjusted analyses, 1-4- and 5-9-year survivors with short compared to long education had statistically significantly higher RRs for GP consultations across all cancer sites (RRs range: 1.14–1.28 and 1.14–1.24, respectively) (Fig. 3, Supplementary Table 3). Furthermore, in stratified analyses, most RRs for GP consultations remained statistically significantly higher for 1-4-year survivors with short compared to long education, irrespective of comorbidity, stage, or treatment (Fig. 3). Similarly, 1-4- and 5-9-year survivors with medium education had statistically significantly higher RRs for GP consultations than survivors with long education (RRs range: 1.10–1.16 and 1.05–1.14, respectively) (Supplementary Table 3). No association was found between educational level and number of hospital contacts (Fig. 3, Supplementary Table 4). In analyses on the use of acute healthcare services, 1-4- and 5-9-year survivors with short education had statistically significantly higher RRs for acute healthcare contacts compared to survivors with long education, regardless of cancer type (RRs range: 1.24–1.35 and 1.22–1.33, respectively) (Fig. 3, Supplementary Table 5). One-to-four- and 5-9-year survivors with short compared to long education had statistically significantly lower RRs for PPS consultations (RRs range: 0.70–0.79 and 0.67–0.76, respectively) (Fig. 3, Supplementary Table 6). Similar patterns of increased RRs for acute healthcare contacts and lower RRs for consultations with PPS among survivors with medium compared to long education was observed (Supplementary Tables 5, Supplementary Table 6).

## Discussion

In this large population-based cohort study, survivors after breast, prostate, lung, and colon cancer had considerably more healthcare contacts than matched cancer-free individuals up to 10 years after diagnosis. Regardless of cancer type, survivors with short education had more GP consultations than survivors with long education, even when taking comorbidity, disease stage, time since diagnosis, and primary cancer treatment into account. Survivors from breast, prostate, lung, and colon cancer with short education had more acute healthcare contacts than survivors with long education. Oppositely, survivors with short education had fewer PPS consultations than survivors with long education. These findings could be interpreted as indicating more health care needs among cancer survivors with short education but could potentially also suggest differences in health-seeking behaviors and/or referral patterns between survivors with short and long education.

Consistent with previous studies, we found elevated use of GP consultations among cancer survivors compared to cancer-free populations [9, 11–13], even several years after diagnosis [7, 8, 10]. Possible explanations could be that cancer treatment is associated with the development of late effects after cancer, that preexisting comorbidities may be affected by the cancer treatment, and that having had cancer makes people aware of symptoms that might prompt more visits to their GP. Results from the present study also show that cancer survivors use more hospital contacts than cancer-free individuals. Although many cancer patients finalize their active treatment within the first year after diagnosis, some will experience relapse, and those who receive palliative care as first-line treatment might receive life-long therapy. This might account for some of the excess use of hospital contacts among cancer survivors compared to cancer-free individuals. Cancer survivors have a higher risk for somatic and psychological morbidities than individuals without a history of cancer [3] and consequently healthcare systems need to prepare for the increasing number of people living with and after cancer and their health care needs.

To our knowledge, only two studies have examined the association between SEP and healthcare use among cancer survivors [4, 17]. One study among 1316 breast cancer survivors, 5–15 years after diagnosis, found that survivors with short or medium compared with long education were more likely to have been in contact with a physician within the past 3 months but no estimates reached statistical significance and healthcare use was self-reported [4]. The other study examined differences in self-reported healthcare use during the past 12 months at 5-year follow-up among 1718 colorectal cancer survivors and showed that survivors with short compared to long education had more frequent GP visits [17]. In the present study there were no differences in hospital contacts between survivors with short and long education for both 1-4- and 5-9-year survivors. These results could in part be explained by the organization of the Danish healthcare system, where GPs have a gatekeeper function for accessing hospital-based services, except in emergency cases [33]. The access to hospitals is thereby limited to patients whom the GP finds need to be treated at the hospital. However, cancer survivors with short compared to long education had more GP consultations, more acute healthcare contacts, and less consultations with PPS in the present study. These results are in accordance with the findings of previous studies, which show that low SEP is related to overall increased healthcare use, reflecting the socioeconomic gradient in health in general [14, 15], and other studies which show that people with low SEP use more emergency and acute healthcare services, while people with high SEP more often use specialty care services [34–38]. Furthermore, high SEP is associated with higher health literacy [16, 39] and better healthcare navigation [16] reflecting that healthcare-seeking behaviors differ by SEP. Cancer survivors with short compared to long education had more acute healthcare contacts. Unplanned emergency healthcare contacts are in many cases unfavorable in terms of continuity of care, and the patients’ relationship with care providers may be less optimal due to incomplete knowledge of the patients’ medical history [34, 36, 40]. Suboptimal use of the healthcare systems might therefore reinforce health disparities between socioeconomic groups. Cancer survivors with short education might benefit from interventions to enhance health literacy and support them in healthcare navigation. Furthermore there may be a need for more targeted focus from GPs to address cancer survivors’ specific needs, in order to potentially avoid a later need for acute healthcare. The socioeconomic gradient in the prevalence of multimorbidity [41] might be another factor that explains why cancer survivors with short education had more GP consultations and acute contacts. Chronic conditions are more likely to be less optimally treated [42, 43] and to progress in people with low compared to high SEP [44]. Thus, cancer survivors with short education might struggle with more health problems, and a greater treatment burden [45, 46], which may lead to more healthcare contacts [41].

### Strengths and limitations

The strengths of our study included a large-scale, population-based cohort design, based on information from the Danish national registers, which, uniquely [23, 47], have close to complete cancer registration and comprehensive information about stage of disease, treatment, comorbidity, healthcare use, and education. We included cancer survivors from 1 and up to 10 years after diagnosis, hence including both short- and long-term survivors, and we had virtually no loss to follow-up. Furthermore, we excluded cancer-related follow-up visits to the hospitals, as to only include need-based healthcare services and not contacts related to pre-planned follow-up programs.

Nevertheless, some limitations deserve consideration. First, we only included healthcare services administered by medical doctors, while we could not include supportive care such as physiotherapists, psychologists, or dentists which is also very relevant to the survivor population. Second, we excluded 27% of prostate cancer survivors from the study population because of missing information on stage at diagnosis. There may be an overweight without registered TNM stage among older men with advanced stage, which might have led to an underestimation of healthcare use among prostate cancer survivors. Third, our results could be affected by a better medical surveillance of cancer survivors than cancer-free individuals.

## Conclusion

Survivors of breast, prostate, lung, and colon cancer use more healthcare services than individuals without cancer, both in terms of GP, acute, and hospital services up to 10 years after diagnosis. The number of GP consultations and acute healthcare contacts was highest while the number of consultations with PPS was lowest among survivors with short education. No differences were found in the number of hospital contacts between survivors with different educational levels. The results suggest an imbalance in symptom burden, healthcare-seeking behaviors, and received health care between survivors with short and long education. To develop interventions with the aim of reducing socioeconomic disparities in healthcare use, there is a need for improved understanding of the mechanisms behind healthcare-seeking behaviors among cancer survivors and their specific health care needs. If cancer survivors’ needs are met with timely and appropriate healthcare and with guidance for vulnerable cancer survivors on how to navigate the healthcare system, we may ensure that all patients get correct care in time and thereby prevent avoidable emergency care.

## Supplementary Information

**Additional file 1: Supplementary Table 1. **Categorization of disease stage and received treatment for breast, prostate, lung, and colon cancer. **Supplementary Table 2.** Results from Poisson regression analyses examining acute and PPS contacts between CS and cancer-free individuals. **Supplementary Table 3.** Results from Poisson regression analyses examining GP consultations between cancer survivors with different educational levels. **Supplementary Table 4.** Results from Poisson regression analyses examining hospital contacts between cancer survivors with different educational levels. **Supplementary Table 5.** Results from Poisson regression analyses examining acute contacts between cancer survivors with different educational levels. **Supplementary Table 6.** Results from Poisson regression analyses examining PPS consultations between cancer survivors with different educational levels.

## Data Availability

The supporting data are available from the Danish Health Data Authority but restrictions apply to the availability of these data, and so are not publicly available. The data that support the findings of this study are available for collaborative research projects upon reasonable request to PI (Susanne Oksbjerg Dalton) and with permission from the Danish Health Data Authority.

## Abbreviations

GP: General practitioner
SEP: Socioeconomic position
RR: Rate ratio
CPR: Central population registry
P-Y: Person years
PPS: Private practicing specialists
CS: Cancer survivors

## References

[1] Bluethmann SM, Mariotto AB, Rowland JH (2016). Anticipating the “Silver Tsunami”: Prevalence Trajectories and Comorbidity Burden among Older Cancer Survivors in the United States. Cancer Epidemiol Biomarkers Prev.

[2] Shapiro CL (2018). Cancer Survivorship. N Engl J Med.

[3] Kjaer TK, Andersen EAW, Winther JF, Bidstrup PE, Borre M, Møller H (2019). Long-term somatic disease risk in adult danish Cancer survivors. JAMA Oncol.

[4] Peuckmann VP, Ekholm O, Sjogren P, Rasmussen NK, Christiansen P, Moller S (2009). Health care utilisation and characteristics of long-term breast cancer survivors: nationwide survey in Denmark. Eur J cancer (Oxford England: 1990).

[5] Treanor C, Santin O, Mills M, Donnelly M (2013). Cancer survivors with self-reported late effects: their health status, care needs and service utilisation. Psycho-oncology.

[6] Silvia J, Hans S, Juliana D (2000). Timescale of evolution of late radiation injury after postoperative radiotherapy of breast cancer patients. Int J Radiation Oncology*Biology*Physics.

[7] Brandenbarg D, Roorda C, Groenhof F, de Bock GH, Berger MY, Berendsen AJ. Primary healthcare use during follow-up after curative treatment for colorectal cancer. Eur J Cancer Care. 2017;26(3). 10.1111/ecc.12581 27726218

[8] Heins M, Schellevis F, Rijken M, van der Hoek L, Korevaar J (2012). Determinants of increased primary health care use in cancer survivors. J Clin Oncol.

[9] Joly F, Henry-Amar M, Arveux P (1996). Late psychosocial sequelae in Hodgkin’s disease survivors: a french population-based case-control study. J Clin Oncol.

[10] Khan NF, Watson E, Rose PW (2011). Primary care consultation behaviours of long-term, adult survivors of cancer in the UK. Br J Gen practice: J Royal Coll Gen Practitioners.

[11] Mols F, Helfenrath KA, Vingerhoets AJ, Coebergh JW, van de Poll-Franse LV (2007). Increased health care utilization among long-term cancer survivors compared to the average dutch population: a population-based study. Int J Cancer.

[12] Nord C, Mykletun A, Thorsen L, Bjoro T, Fossa SD (2005). Self-reported health and use of health care services in long-term cancer survivors. Int J Cancer.

[13] Snyder CF, Frick KD, Peairs KS, Kantsiper ME, Herbert RJ, Blackford AL (2009). Comparing care for breast cancer survivors to non-cancer controls: a five-year longitudinal study. J Gen Intern Med.

[14] Droomers M, Westert GP (2004). Do lower socioeconomic groups use more health services, because they suffer from more illnesses?. Eur J Public Health.

[15] Grosse Frie K, Eikemo TA, von dem Knesebeck O (2010). Education and self-reported health care seeking behaviour in european welfare regimes: results from the european Social Survey. Int J Public Health.

[16] Jansen T, Rademakers J, Waverijn G, Verheij R, Osborne R, Heijmans M (2018). The role of health literacy in explaining the association between educational attainment and the use of out-of-hours primary care services in chronically ill people: a survey study. BMC Health Serv Res.

[17] Thong MSY, Boakye D, Jansen L, Martens UM, Chang-Claude J, Hoffmeister M (2021). Comorbidities, rather than older Age, are strongly Associated with higher utilization of Healthcare in Colorectal Cancer Survivors. J Natl Compr Cancer Network: JNCCN.

[18] Larønningen S, Ferlay J, Beydogan H, Bray F, Engholm G, Ervik M et al. NORDCAN: Cancer Incidence, Mortality, Prevalence and Survival in the Nordic Countries, Version 9.2 (23.06.2022). Association of the Nordic Cancer Registries. Cancer Registry of Norway. 2022 [Available from: https://nordcan.iarc.fr/.

[19] Christiansen P, Ejlertsen B, Jensen MB, Mouridsen H (2016). Danish breast Cancer Cooperative Group. Clin Epidemiol.

[20] Nguyen-Nielsen M, Hoyer S, Friis S, Hansen S, Brasso K, Jakobsen EB (2016). The danish prostate Cancer Database. Clin Epidemiol.

[21] Jakobsen E, Rasmussen TR (2016). The danish Lung Cancer Registry. Clin Epidemiol.

[22] Ingeholm P, Gogenur I, Iversen LH (2016). Danish colorectal Cancer Group Database. Clin Epidemiol.

[23] Thygesen LC, Daasnes C, Thaulow I, Bronnum-Hansen H (2011). Introduction to danish (nationwide) registers on health and social issues: structure, access, legislation, and archiving. Scand J Public Health.

[24] Pedersen CB (2011). The danish Civil Registration System. Scand J Public Health.

[25] Galobardes B, Shaw M, Lawlor DA, Lynch JW, Davey Smith G (2006). Indicators of socioeconomic position (part 1). J Epidemiol Commun Health.

[26] Schmidt M, Schmidt SAJ, Adelborg K, Sundbøll J, Laugesen K, Ehrenstein V (2019). The danish health care system and epidemiological research: from health care contacts to database records. Clin Epidemiol.

[27] Pedersen KM, Andersen JS, Søndergaard J (2012). General practice and primary health care in Denmark. J Am Board Family Medicine: JABFM.

[28] Andersen JS, Olivarius Nde F, Krasnik A (2011). The danish National Health Service Register. Scand J Public Health.

[29] Schmidt M, Schmidt SA, Sandegaard JL, Ehrenstein V, Pedersen L, Sørensen HT (2015). The danish National Patient Registry: a review of content, data quality, and research potential. Clin Epidemiol.

[30] Charlson ME, Pompei P, Ales KL, MacKenzie CR (1987). A new method of classifying prognostic comorbidity in longitudinal studies: development and validation. J Chronic Dis.

[31] Chiou SH, Xu G, Yan J, Huang C. -YJapa. Regression Modeling for Recurrent Events Using R Package reReg. 2021. 10.18637/jss.v105.i05 PMC10997344 38586564

[32] R Development Core Team (2022). R: a language and environment for statistical computing.

[33] Mooney G. The Danish health care system: it ain't broke... so don't fix it. Health policy (Amsterdam, Netherlands). 2002;59(2):161-71. 10.1016/s0168-8510(01)00205-6 11755997

[34] Drummond N, McConnachie A, O’Donnell CA, Moffat KJ, Wilson P, Ross S (2000). Social variation in reasons for contacting general practice out-of-hours: implications for daytime service provision?. Br J Gen practice: J Royal Coll Gen Practitioners.

[35] Glazier RH, Agha MM, Moineddin R, Sibley LM (2009). Universal health insurance and equity in primary care and specialist office visits: a population-based study. Ann Fam Med.

[36] Kangovi S, Barg FK, Carter T, Long JA, Shannon R, Grande D (2013). Understanding why patients of low socioeconomic status prefer hospitals over ambulatory care. Health Aff (Millwood).

[37] Salisbury C, Trivella M, Bruster S (2000). Demand for and supply of out of hours care from general practitioners in England and Scotland: observational study based on routinely collected data. BMJ (Clinical research ed).

[38] Willems S, Peersman W, De Maeyer P, Buylaert W, De Maeseneer J, De Paepe P (2013). The impact of neighborhood deprivation on patients’ unscheduled out-of-hours healthcare seeking behavior: a cross-sectional study. BMC Fam Pract.

[39] Svendsen MT, Bak CK, Sorensen K, Pelikan J, Riddersholm SJ, Skals RK (2020). Associations of health literacy with socioeconomic position, health risk behavior, and health status: a large national population-based survey among danish adults. BMC Public Health.

[40] Huibers L, Giesen P, Wensing M, Grol R (2009). Out-of-hours care in western countries: assessment of different organizational models. BMC Health Serv Res.

[41] Frolich A, Ghith N, Schiotz M, Jacobsen R, Stockmarr A (2019). Multimorbidity, healthcare utilization and socioeconomic status: a register-based study in Denmark. PLoS ONE.

[42] Ho PM, Rumsfeld JS, Masoudi FA, McClure DL, Plomondon ME, Steiner JF (2006). Effect of medication nonadherence on hospitalization and mortality among patients with diabetes mellitus. Arch Intern Med.

[43] Oates GR, Juarez LD, Hansen B, Kiefe CI, Shikany JM (2020). Social Risk factors for Medication Nonadherence: findings from the CARDIA Study. Am J Health Behav.

[44] Mira R, Newton T, Sabbah W (2022). Inequalities in the progress of multiple chronic conditions: a systematic review of longitudinal studies. PLoS ONE.

[45] Haynes RB, McDonald HP, Garg AX (2002). Helping patients follow prescribed treatment: clinical applications. JAMA.

[46] Kunt T, Snoek FJ (2009). Barriers to insulin initiation and intensification and how to overcome them. Int J Clin Pract Supplement..

[47] Gjerstorff ML (2011). The danish Cancer Registry. Scand J Public Health.

