# Supplementary material for: Educational differences in healthcare use among survivors after breast, prostate, lung, and colon cancer – a SEQUEL cohort study
Source: BMC Health Serv Res. 2023 Jun 22;23:674. doi: 10.1186/s12913-023-09683-2 (PMC10286377; doi:10.1186/s12913-023-09683-2)
Supplement: Supplementary file 1 — Additional file 1: Supplementary Table 1. Categorization of disease stage and received treatment for breast, prostate, lung, and colon cancer. Supplementary Table 2. Results from Poisson regression analyses examining acute and PPS contacts between CS and cancer-free individuals. Supplementary Table 3. Results from Poisson regression analyses examining GP consultations between cancer survivors with different educational levels. Supplementary Table 4. Results from Poisson regression analyses examining hospital contacts between cancer survivors with different educational levels. Supplementary Table 5. Results from Poisson regression analyses examining acute contacts between cancer survivors with different educational levels. Supplementary Table 6. Results from Poisson regression analyses examining PPS consultations between cancer survivors with different educational levels. [file 12913_2023_9683_MOESM1_ESM.docx]

**Supplementary Table 1.** Categorization of disease stage and received treatment for breast, prostate, lung, and colon cancer

| **Cancer type** | **Categorization** | | | |
| --- | --- | --- | --- | --- |
|  | **Local/regional stage** | **Advanced stage** | **Curative treatment** | **Palliative treatment** |
| Breast | any tumor size, number of positive lymph nodes, and no distant metastasis | Distant metastasis | multimodality treatment for early-stage disease | treatment for advanced stage disease |
| Prostate | TNM stage with any T, any N, and no M | TNM stage with distant metastasis | prostatectomy, active surveillance, or curatively intended radiotherapy | palliative radiotherapy, endocrine therapy, or watchful waiting |
| Lung | TNM stage at IA, IB, IIA, IIB, or IIIA | TNM stage IIIB, IIIC, IVA, or IVB | curatively intended chemo-, and/or radiotherapy, surgery or neo-/adjuvant therapy | palliative chemo- and/or radiotherapy, and other treatment with palliative intent |
| Colon | UICC (8^th^ edition) stage I, II, or III | UICC (8^th^ edition) stage IV | surgery with curative intent | surgery with palliative intent and chemo-, and/or radiotherapy with palliative intent |

**Supplementary Table 2.** Results from Poisson regression analyses examining acute and PPS contacts between CS and cancer-free individuals

|  | **Acute healthcare contacts** | | | | | |
| --- | --- | --- | --- | --- | --- | --- |
|  | **1-4 years** | | | **5-9 years** | | |
|  | Crude incidence per 100 P-Y | Model 1  RR (95% CI) | Model 2  RR (95% CI) | Crude incidence per 100 P-Y | Model 1  RR (95% CI) | Model 2  RR (95% CI) |
| Breast cancer** | 88 | 1.33 (1.30; 1.36) | 1.36 (1.33; 1.39) | 87 | 1.24 (1.22; 1.27) | 1.27 (1.25; 1.30) |
| Cancer-free individuals | 67 | Ref. | Ref. | 71 | Ref. | Ref. |
| Prostate cancer** | 116 | 1.42 (1.38; 1.46) | 1.51 (1.47; 1.55) | 116 | 1.26 (1.21; 1.32) | 1.31 (1.25; 1.36) |
| Cancer-free individuals | 83 | Ref. | Ref. | 94 | Ref. | Ref. |
| Lung cancer | 283 | 3.92 (3.82; 4.03) | 3.57 (3.47; 3.66) | 185 | 2.37 (2.22; 2.53) | 2.18 (2.04; 2.34) |
| Cancer-free individuals | 73 | Ref. | Ref. | 82 | Ref. | Ref. |
| Colon cancer | 149 | 1.66 (1.56; 1.76) | 1.66 (1.56; 1.77) | 127 | 1.28 (1.23; 1.33) | 1.28 (1.24; 1.33) |
| Cancer-free individuals | 89 | Ref. | Ref. | 96 | Ref. | Ref. |
|  | **Private practicing specialists** | | | | | |
|  | **1-4 years** | | | **5-9 years** | | |
|  | Crude incidence per 100 P-Y | Model 1  RR (95% CI) | Model 2  RR (95% CI) | Crude incidence per 100 P-Y | Model 1  RR (95% CI) | Model 2  RR (95% CI) |
| Breast cancer** | 153 | 1.14 (1.13; 1.16) | 1.13 (1.11; 1.15) | 157 | 1.12 (1.10; 1.14) | 1.11 (1.09; 1.13) |
| Cancer-free individuals | 135 | Ref. | Ref. | 140 | Ref. | Ref. |
| Prostate cancer** | 140 | 1.04 (1.01; 1.07) | 1.03 (1.00; 1.06) | 152 | 1.09 (1.05; 1.13) | 1.08 (1.04; 1.12) |
| Cancer-free individuals | 129 | Ref. | Ref. | 140 | Ref. | Ref. |
| Lung cancer | 129 | 0.97 (0.94; 1.01) | 0.99 (0.95; 1.02) | 144 | 1.04 (0.97; 1.11) | 1.06 (0.99; 1.14) |
| Cancer-free individuals | 133 | Ref. | Ref. | 140 | Ref. | Ref. |
| Colon cancer | 138 | 1.01 (0.98; 1.04) | 1.00 (0.97; 1.03) | 151 | 1.06 (1.02; 1.10) | 1.05 (1.01; 1.09) |
| Cancer-free individuals | 137 | Ref. | Ref. | 142 | Ref. | Ref. |

Cancer-free matched comparison people are the reference for all analyses. PPS, private practicing specialists, CS, cancer survivors, RR, rate ratios, CI, confidence interval, P-Y, person-years.

Model 1: adjusted for age, sex, time since diagnosis/index date, year of diagnosis

Model 2: adjusted for age, sex, time since diagnosis/index date, year of diagnosis, cohabitation status, comorbidity, education

* 5-8 year for survivors for prostate cancer

** Analyses are not adjusted for sex

**Supplementary Table 3.** Results from Poisson regression analyses examining GP consultations between cancer survivors with different educational levels

|  | **GP consultations, RR (95% CI)** | | | | | |
| --- | --- | --- | --- | --- | --- | --- |
|  | **1-4 years** | | | **5-9 years*** | | |
|  | Model 1 | Model 2 | Model 3 | Model 1 | Model 2 | Model 3 |
| **Breast cancer**** | | | | | | |
| Short education | 1.31 (1.28; 1.33) | 1.28 (1.25; 1.30) | 1.28 (1.25; 1.30) | 1.26 (1.23; 1.29) | 1.24 (1.21; 1.27) | 1.24 (1.21; 1.27) |
| Medium education | 1.17 (1.15; 1.18) | 1.16 (1.14; 1.18) | 1.16 (1.14; 1.18) | 1.15 (1.13; 1.17) | 1.15; 1.12; 1.17) | 1.14 (1.12; 1.17) |
| **Prostate cancer**** | | | | | | |
| Short education | 1.18 (1.15; 1.22) | 1.16 (1.12; 1.19) | 1.14 (1.10; 1.18) | 1.17 (1.12; 1.24) | 1.15 (1.10; 1.21) | 1.15 (1.09; 1.22) |
| Medium education | 1.12 (1.10; 1.15) | 1.11 (1.08; 1.13) | 1.11 (1.07; 1.14) | 1.07 (1.03; 1.12) | 1.06 (1.02; 1.10) | 1.05 (1.02; 1.12) |
| **Lung cancer** | | | | | | |
| Short education | 1.20 (1.15; 1.25) | 1.17 (1.12; 1.22) | 1.18 (1.13; 1.23) | 1.18 (1.09; 1.29) | 1.16 (1.07; 1.26) | 1.16 (1.07; 1.26) |
| Medium education | 1.11 (1.07; 1.15) | 1.09 (1.05; 1.14) | 1.10 (1.05; 1.14) | 1.13 (1.05; 1.22) | 1.12 (1.04; 1.21) | 1.12 (1.04; 1.20) |
| **Colon cancer** | | | | | | |
| Short education | 1.21 (1.16; 1.26) | 1.17 (1.12; 1.21) | 1.17 (1.13; 1.22) | 1.16 (1.10; 1.22) | 1.14 (1.08; 1.20) | 1.14 (1.08; 1.20) |
| Medium education | 1.11 (1.08; 1.14) | 1.09 (1.06; 1.12) | 1.10 (1.06; 1.13) | 11.14 (1.09; 1.20) | 1.13 (1.08; 1.18) | 1.13 (1.08; 1.18) |

Long education is the reference for all analyses. GP, general practitioner, RR, rate ratios, CI, confidence interval.

Model 1: adjusted for age, sex, time since diagnosis, year of diagnosis

Model 2: adjusted for age, sex, time since diagnosis, year of diagnosis, cohabitation status, comorbidity, education

Model 3: adjusted for age, sex, time since diagnosis, year of diagnosis, cohabitation status, comorbidity, education, stage at diagnosis

* 5-8 year for survivors for prostate cancer

** Analyses are not adjusted for sex

**Supplementary Table 4.** Results from Poisson regression analyses examining hospital contacts between cancer survivors with different educational levels

|  | **Hospital contacts, RR (95% CI)** | | | | | |
| --- | --- | --- | --- | --- | --- | --- |
|  | **1-4 years** | | | **5-9 years*** | | |
|  | Model 1 | Model 2 | Model 3 | Model 1 | Model 2 | Model 3 |
| **Breast cancer**** | | | | | | |
| Short education | 1.06 (1.04; 1.09) | 1.04 (1.01; 1.07) | 1.01 (0.98; 1.03) | 1.05 (1.02; 1.10) | 1.02 (0.98; 1.07) | 1.01 (0.97; 1.06) |
| Medium education | 1.06 (1.03; 1.08) | 1.05 (1.02; 1.07) | 1.03 (1.01; 1.05) | 1.05 (1.02; 1.09) | 1.04 (1.00; 1.08) | 1.03 (0.99; 1.07) |
| **Prostate cancer**** | | | | | | |
| Short education | 1.08 (1.02; 1.14) | 1.05 (1.00; 1.11) | 1.03 (0.97; 1.10) | 1.02 (0.92; 1.13) | 1.00 (0.91; 1.10) | 0.96 (0.85; 1.08) |
| Medium education | 1.10 (1.05; 1.14) | 1.08 (1.03; 1.12) | 1.06 (1.01; 1.12) | 0.99 (0.91; 1.07) | 0.98 (0.90; 1.06) | 0.97 (0.88; 1.07) |
| **Lung cancer** | | | | | | |
| Short education | 0.95 (0.90; 1.00) | 0.94 (0.89; 1.00) | 0.97 (0.92; 1.02) | 0.91 (0.79; 1.05) | 0.89 (0.78; 1.03) | 0.90 (0.79; 1.04) |
| Medium education | 0.98 (0.93; 1.04) | 0.98 (0.93; 1.03) | 0.99 (0.94; 1.04) | 0.95 (0.82; 1.09) | 0.93 (0.81; 1.07) | 0.94 (0.82; 1.08) |
| **Colon cancer** | | | | | | |
| Short education | 0.95 (0.89; 1.02) | 0.92 (0.86; 0.98) | 0.96 (0.90; 1.02) | 0.93 (0.83; 1.03) | 0.89 (0.80; 0.99) | 0.90 (0.81; 1.00) |
| Medium education | 0.97 (0.92; 1.03) | 0.95 (0.90; 1.01) | 1.00 (0.95; 1.05) | 1.01 (0.90; 1.12) | 0.99 (0.89; 1.10) | 1.00 (0.90; 1.11) |

Long education is the reference for all analyses. RR, rate ratios, CI, confidence interval.

Model 1: adjusted for age, sex, time since diagnosis, year of diagnosis

Model 2: adjusted for age, sex, time since diagnosis, year of diagnosis, cohabitation status, comorbidity, education

Model 3: adjusted for age, sex, time since diagnosis, year of diagnosis, cohabitation status, comorbidity, education, stage at diagnosis

* 5-8 year for survivors for prostate cancer

** Analyses are not adjusted for sex

**Supplementary Table 5.** Results from Poisson regression analyses examining acute contacts between cancer survivors with different educational levels

|  | **Acute contacts, RR (95% CI)** | | | | | |
| --- | --- | --- | --- | --- | --- | --- |
|  | **1-4 year** | | | **5-9 year*** | | |
|  | Model 1 | Model 2 | Model 3 | Model 1 | Model 2 | Model 3 |
| **Breast cancer**** | | | | | | |
| Short education | 1.44 (1.35; 1.54) | 1.38 (1.29; 1.47) | 1.35 (1.26; 1.45) | 1.32 (1.26; 1.40) | 1.28 (1.21; 1.35) | 1.26 (1.20; 1.33) |
| Medium education | 1.17 (1.12; 1.22) | 1.16 (1.11; 1.21) | 1.15 (1.10; 1.20) | 1.13 (1.08; 1.19) | 1.13 (1.08; 1.18) | 1.12 (1.06; 1.17) |
| **Prostate cancer**** | | | | | | |
| Short education | 1.34 (1.25; 1.44) | 1.27 (1.19; 1.37) | 1.26 (1.15; 1.38) | 1.30 (1.25; 1.35) | 1.24 (1.19; 1.29) | 1.22 (1.18; 1.27) |
| Medium education | 1.17 (1.10; 1.35) | 1.13 (1.06; 1.21) | 1.14 (1.04; 1.24) | 1.10 (1.06; 1.14) | 1.07 (1.04; 1.11) | 1.06 (1.02; 1.09) |
| **Lung cancer** | | | | | | |
| Short education | 1.26 (1.18; 1.34) | 1.21 (1.14; 1.30) | 1.24 (1.16; 1.33) | 1.28 (1.06; 1.54) | 1.24 (1.03; 1.50) | 1.25 (1.04; 1.50) |
| Medium education | 1.14 (1.07; 1.21) | 1.11 (1.05; 1.19) | 1.13 (1.06; 1.20) | 1.07 (0.90; 1.27) | 1.05 (0.89; 1.25) | 1.06 (0.89; 1.25) |
| **Colon cancer** | | | | | | |
| Short education | 1.43 (1.16; 1.75) | 1.32 (1.11; 1.57) | 1.35 (1.14; 1.60) | 1.39 (1.25; 1.55) | 1.33 (1.19; 1.48) | 1.33 (1.20; 1.48) |
| Medium education | 1.10 (1.04; 1.17) | 1.06 (1.00; 1.13) | 1.09 (1.03; 1.16) | 1.24 (1.13; 1.36) | 1.21 (1.10; 1.32) | 1.21 (1.10; 1.33) |

Long education is the reference for all analyses. RR, rate ratios, CI, confidence interval.

Model 1: adjusted for age, sex, time since diagnosis, year of diagnosis

Model 2: adjusted for age, sex, time since diagnosis, year of diagnosis, cohabitation status, comorbidity, education

Model 3: adjusted for age, sex, time since diagnosis, year of diagnosis, cohabitation status, comorbidity, education, stage at diagnosis

* 5-8 year for survivors for prostate cancer

** Analyses are not adjusted for sex

**Supplementary Table 6.** Results from Poisson regression analyses examining PPS consultations between cancer survivors with different educational levels

|  | **Private practicing specialists, RR (95% CI)** | | | | | |
| --- | --- | --- | --- | --- | --- | --- |
|  | **1-4 year** | | | **5-9 year*** | | |
|  | Model 1 | Model 2 | Model 3 | Model 1 | Model 2 | Model 3 |
| **Breast cancer**** | | | | | | |
| Short education | 0.71 (0.69; 0.74) | 0.71 (0.68; 0.74) | 0.72 (0.69; 0.75) | 0.72 (0.68; 0.75) | 0.71 (0.68; 0.75) | 0.72 (0.68; 0.75) |
| Medium education | 0.89 (0.86; 0.91) | 0.88 (0.86; 0.91) | 0.89 (0.86; 0.92) | 0.91 (0.88; 0.94) | 0.91 (0.88; 0.95) | 0.91 (0.87; 0.94) |
| **Prostate cancer**** | | | | | | |
| Short education | 0.74 (0.69; 0.79) | 0.74 (0.69; 0.79) | 0.79 (0.74; 0.86) | 0.75 (0.67; 0.84) | 0.75 (0.67; 0.84) | 0.74 (0.65; 0.84) |
| Medium education | 0.88 (0.84; 0.93) | 0.88 (0.84; 0.93) | 0.92 (0.87; 0.97) | 0.89 (0.82; 0.96) | 0.89 (0.82; 0.96) | 0.89 (0.81; 0.98) |
| **Lung cancer** | | | | | | |
| Short education | 0.74 (0.66; 0.82) | 0.74 (0.66; 0.83) | 0.73 (0.66; 0.82) | 0.78 (0.64; 0.96) | 0.77 (0.63; 0.93) | 0.76 (0.63; 0.92) |
| Medium education | 0.89 (0.81; 0.99) | 0.89 (0.81; 0.99) | 0.89 (0.80; 0.98) | 0.95 (0.81; 1.12) | 0.94 (0.80; 1.11) | 0.94 (0.80; 1.10) |
| **Colon cancer** | | | | | | |
| Short education | 0.71 (0.65; 0.76) | 0.71 (0.65; 0.76) | 0.70 (0.65; 0.76) | 0.66 (0.59; 0.75) | 0.67 (0.59; 0.76) | 0.67 (0.59; 0.76) |
| Medium education | 0.86 (0.81; 0.92) | 0.86 (0.81; 0.92) | 0.86 (0.80; 0.92) | 0.85 (0.77; 0.93) | 0.85 (0.77; 0.93) | 0.85 (0.77; 0.93) |

Long education is the reference for all analyses. PPS, private practicing specialists, RR, rate ratios, CI, confidence interval.

Model 1: adjusted for age, sex, time since diagnosis/, year of diagnosis

Model 2: adjusted for age, sex, time since diagnosis, year of diagnosis, cohabitation status, comorbidity, education

Model 3: adjusted for age, sex, time since diagnosis, year of diagnosis, cohabitation status, comorbidity, education, stage at diagnosis

* 5-8 year for survivors for prostate cancer

** Analyses are not adjusted for sex
